# Association between household solid fuel use and dual sensory impairment in a Chinese population: a retrospective cohort study

**DOI:** 10.3389/fpubh.2025.1439673

**Published:** 2025-06-10

**Authors:** Yaolei Du, Mengnan Wu, Mansha He

**Affiliations:** Department of Ophthalmology, Guangzhou Aier Eye Hospital Affiliated to Jinan University, Guangzhou, China

**Keywords:** dual sensory impairment, household solid fuels, risk, CHARLS database, household air pollution

## Abstract

**Aim:**

Dual sensory impairment (DSI) is more harmful than a single visual impairment or hearing impairment. We aimed to explore the relationship between household fuel use and the risk of DSI in the middle-aged and older adult Chinese population.

**Methods:**

Data from the China Health and Retirement Longitudinal Study (CHARLS) 2015 of 8,083 participants aged ≥45 years were used, followed up to 2018. Household fuels include heating fuels and cooking fuels. Participants were divided into four groups based on the type of household fuel use at baseline (2015) and during follow-up (2018) (baseline and follow-up): clean fuels and clean fuels, clean fuels and solid fuels, solid fuels and clean fuels, and solid fuels and solid fuels. Logistic regression models were used to assess the association between household fuel use and the risk of DSI, and odds ratio (OR) with 95% confidence interval (CI) was utilized to evaluate the association.

**Results:**

Of these 8,083 participants, 886 (10.96%) had hearing impairment, 2,361 (29.21%) had visual impairment, and 505 (6.25%) had DSI. The use of solid fuels at baseline (OR = 1.23, 95%CI: 1.02–1.49) was associated with a higher risk of DSI compared to the use of clean fuels. People in the clean fuels and solid fuels group (OR = 1.50, 95%CI: 1.04–2.16) and the solid fuels and solid fuels group (OR = 1.38, 95%CI: 1.10–1.73) were linked to an increased risk of DSI compared to people in the clean fuels and clean fuels group, whereas no significant difference was observed in the effect on DSI between people in the solid fuels and clean fuels group and the clean fuels and clean fuels group (*p* = 0.99). Subgroup analysis demonstrated that males in the solid fuels and clean fuels group (OR = 0.60, 95%CI: 0.39–0.92) had a lower risk of DSI compared to those in the solid fuels and solid fuels group.

**Conclusion:**

Household solid fuel use is associated with an increased risk of DSI in middle-aged and older Chinese people, and promoting the use of clean fuels is beneficial in reducing the burden of DSI.

## Introduction

Dual sensory impairment (DSI) is defined as having both visual impairment and hearing impairment. Visual impairment and hearing impairment are very common among older people and increase with age (1). According to the World Health Organization (WHO), about 1.5 billion people globally were influenced by hearing impairment and 2.2 billion people by visual impairment and in 2019 (2, 3). The physiological decline caused by visual impairment and hearing impairment can significantly affect the quality of life, mental health, and economic level of the older adult population (4, 5). In addition, there is a synergistic interaction between visual impairment and hearing impairment (6), which exposes people with DSI to more serious adverse health outcomes (7). DSI poses a public health challenge by contributing to high hospitalization rates and high healthcare costs (8).

Exposure to indoor air pollution is a significant contributor to health problems (9). Indoor air pollution from the burning of solid fuels for cooking and heating is responsible for a significant portion of the global burden of death and disease (10). In China, approximately 46% of households are reported to use solid fuels for energy (11). Some previous studies have explored the association between indoor air pollution and visual impairment and hearing impairment, respectively (12–14). People exposed to high levels of nitrogen dioxide (NO), carbon monoxide (CO), and PM2.5 had a significantly higher incidence of hearing damage (12, 13). High levels of PM2.5 and CO from solid fuel use can directly or indirectly cause eye health problems (14). However, the effect of indoor air pollution on the risk of DSI is unclear and it is not clear whether exposure to indoor air pollution would be more likely to cause DSI in people who already have a single hearing or visual impairment. Thus, the aim of this study was to explore the impact of household solid fuel use on the risk of DSI based on data from a nationally representative cohort.

## Methods

### Study design and participants

This retrospective cohort study was based on data from the China Health and Retirement Longitudinal Study (CHARLS). CHARLS is a nationally representative longitudinal survey of people aged 45 years and older in China to assess the social, economic, and health status of community residents (15). CHARLS selected the sample through multi-stage probability sampling, with the final sample drawn from 150 districts in 28 provinces. Baseline data for the participants were collected between June 2011 and March 2012, and three waves of follow-up were conducted in 2013, 2015, and 2018, with face-to-face computer-assisted personal interviews and physical measurements of participants (blood samples were taken every two follow-up periods). This study used data from 2015 and 2018, with the 2015 data as baseline information and the 2018 data as outcome information. The inclusion criteria were as follows: (1) people with complete information on visual function and hearing function in 2015 and 2018; and (2) people with complete information on heating fuels and cooking fuels in 2015 and 2018. Participants with a baseline diagnosis of DSI or missing information on key covariates (e.g., smoking) were excluded. The study protocol of CHARLS was approved by the ethics committee of Peking University and written informed consent was obtained from each participant. This study used de-identified data from publicly available databases and was exempt from ethical review by a local hospital.

### Outcomes

The outcome of this study was the occurrence of DSI during follow-up. DSI was defined as having both visual impairment and hearing impairment at the 2018 follow-up endpoint. Visual impairment was assessed through a self-reported visual status questionnaire (includes near and distant visual status), with possible responses to these questions being “poor,” “fair,” “good,” “very good,” and “excellent.” Participants who reported “poor” on these questions were considered to have visual impairment. Hearing impairment was evaluated by a self-reported hearing status questionnaire, with response options for this question being “poor,” “fair,” “good,” “very good,” and “excellent.” Participants who answered “poor” to this question were classified as having hearing impairment.

### Assessment of household fuels

Household energy sources were evaluated through a questionnaire in 2015 and 2018, and the main energy sources included heating fuels and cooking fuels (16, 17). The specific categories of solid and clean fuels were: solid fuels [cooking (wood burning, coal, solid charcoal, crop residue); heating (wood burning, coal, solid charcoal, crop residue)]; clean fuels [cooking (liquefied petroleum gas, natural gas, electric, marsh gas); heating (liquefied petroleum gas, natural gas, electric, municipal heating, solar energy)]. Those using both clean fuels for heating and cooking were categorized in the clean fuels group, while others (solid heating fuels and clean cooking fuels, or vice versa; both solid fuels for heating and cooking) were categorized in the solid fuels group.

### Covariates

Data on participants were collected including age, gender (male, female), education (illiterate, primary school or below, middle school or above, unknown), marital status (married, other), place of residence (urban, rural), type of house structure (reinforced concrete or masonry structure, other), income (<mean, ≥mean, unknown), body mass index (BMI) (<24 kg/m^2^, ≥24 kg/m^2^, unknown), self-assessed health status (very good, good, fair, poor, very poor, unknown), night sleep duration (<7 h, 7–9 h, >9 h, unknown), smoking (never, former, now), drinking (never, <1 time/month, ≥1 time/month, unknown), hypertension (no, yes, unknown), diabetes (no, yes, unknown), dyslipidemia (no, yes, unknown), cardiovascular disease (CVD) (no, yes, unknown), chronic lung diseases (no, yes, unknown), arthritis or rheumatism (no, yes, unknown), cancer (no, yes, unknown), estimated glomerular filtration rate (eGFR), and sensory disturbance frequency. Hypertension, diabetes, dyslipidemia, CVD, chronic lung diseases, arthritis or rheumatism, and cancer were determined by self-reported physician diagnoses.

### Statistical analysis

Continuous variables were normally distributed and expressed as mean and standard deviation (SD). Categorical variables were expressed as frequency and percentage [*n* (%)]. The student *t*-test was used for comparisons between continuous variables, and the chi-square test or Fisher exact test was used for comparisons between categorical variables.

Covariates were screened using the Adaptive Best-Subset Selection (ABESS) method based on the Generalized Information Criterion (GIC), and covariate screening was performed via the “abbess” R package (version 0.4.8) (Supplementary Table S1) (18). In the abbess algorithm, covariates with coefficients β not 0 were included in the optimal subset. The variance inflation factor (VIF) was applied to evaluate the linear relationship between the covariates, with VIF < 10 indicating the absence of multicollinearity (Supplementary Table S2). The subset of the best covariates after screening were age, gender, place of residence, income, BMI, self-assessed health status, night sleep duration, diabetes, chronic lung diseases, and sensory disturbance. Univariable and multivariable logistic regression models were used to assess the impact of household fuel use on DSI, and odds ratio (OR) with 95% confidence interval (CI) was utilized to evaluate the association. The Hosmer-Lemeshow test was used to assess the goodness-of-fit of the model, and *p* > 0.05 indicates a good consistency of the model fit. In addition, we analyzed the impact of changes in household fuel use during the follow-up period on DSI. Participants were divided into four groups: clean fuel use at both baseline and follow-up (clean fuels and clean fuels); clean fuel at baseline and solid fuel use at follow-up (clean fuels and solid fuels); solid fuel use at baseline and clean fuel in follow-up (solid fuels and clean fuels); solid fuel use at both baseline and follow-up (solid fuels and solid fuels). The impact of household fuel use on DSI was further analyzed according to different subgroups of the population. Statistical analyses were performed by R version 4.2.3 software (Institute for Statistics and Mathematics, Vienna, Austria). *p* < 0.05 (two-tailed) was considered statistically significant.

## Results

### Participant’s characteristics

**Figure 1:**
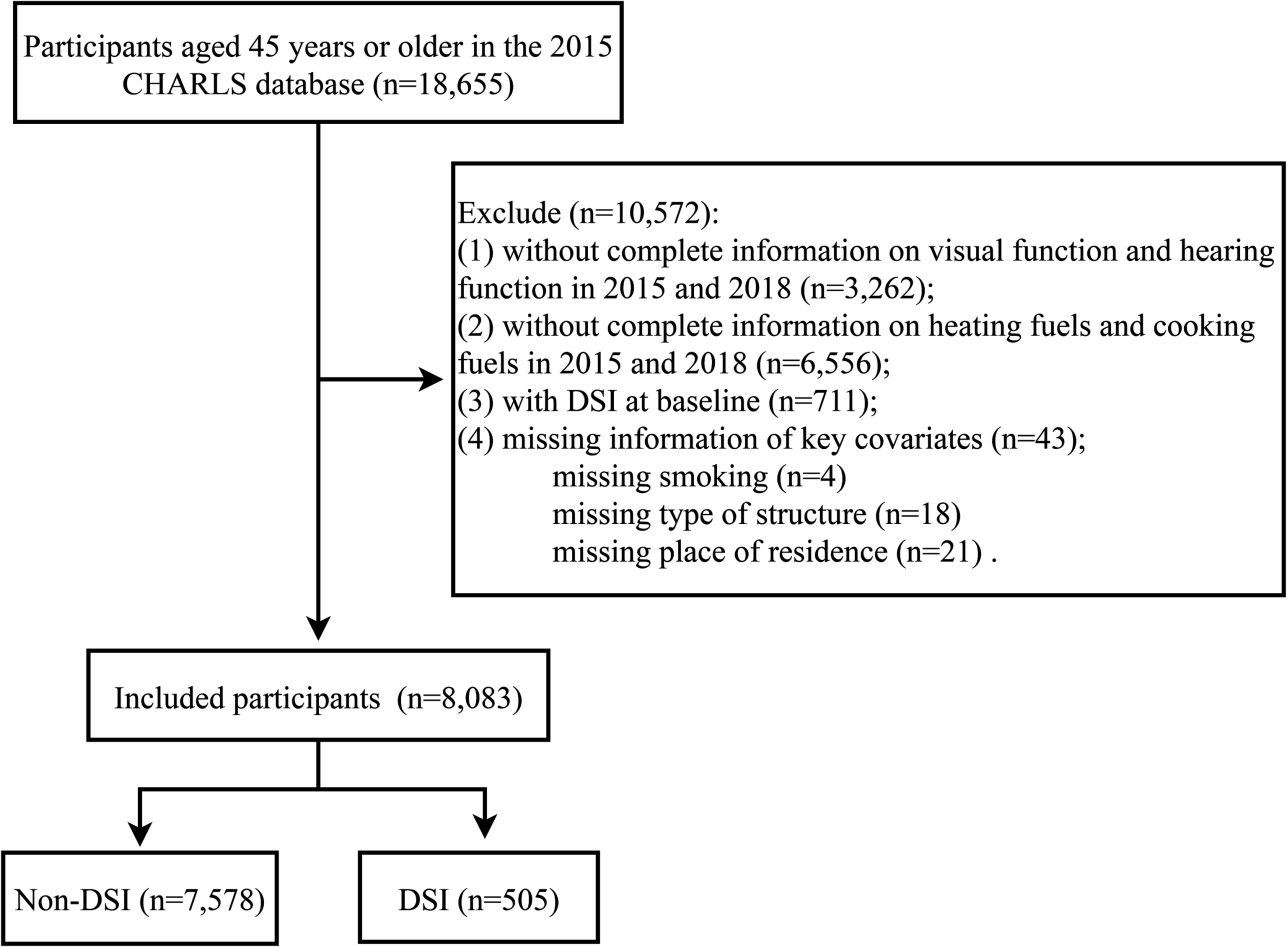
Flowchart for screening the study population.

A total of 18,655 participants were recorded in the 2015 CHARLS database, of which 8,083 participants without DSI at baseline were included in this study (Figure 1). The baseline characteristics of participants according to household fuel use at baseline were listed in Table 1. The characteristics of participants based on household fuel use at baseline and during follow-up were shown in Supplementary Table S3. The mean age of participants was 60.39 (SD 9.79) years and 4,212 (52.11%) participants were male. There were 5,796 (71.71%) participants living in rural areas and 2,287 (28.29%) in urban areas. There were 3,905 (48.31%) participants who stayed on clean fuels use, 2,256 (27.91%) participants who stayed on solid fuels use, 510 (6.31%) participants who used clean fuels at baseline and solid fuels at follow-up, and 1,412 (17.47%) participants who used solid fuels at baseline and clean fuels at follow-up. At the follow-up endpoint of this study, 886 (10.96%) patients had hearing impairment, 2,361 (29.21%) patients had visual impairment, and 505 (6.25%) patients had DSI.

**Table 1:** Baseline characteristics of participants according to household fuels at baseline (2015).

Variables	Total (*N* = 8,083)	Clean fuels (*N* = 4,415)	Solid fuels (*N* = 3,668)	*P*
Age, years, Mean (±SD)	60.39 (±9.79)	59.67 (±9.78)	61.27 (±9.73)	<0.001
Age, years, *n* (%)				<0.001
<65	5,556 (68.74)	3,159 (71.55)	2,397 (65.35)	
≥65	2,527 (31.26)	1,256 (28.45)	1,271 (34.65)	
Gender, *n* (%)				0.659
Male	4,212 (52.11)	2,311 (52.34)	1,901 (51.83)	
Female	3,871 (47.89)	2,104 (47.66)	1,767 (48.17)	
Education, *n* (%)				<0.001
Illiterate	1,370 (16.95)	590 (13.36)	780 (21.26)	
Primary school or below	2,823 (34.93)	1,442 (32.66)	1,381 (37.65)	
Middle school or above	2,424 (29.99)	1,490 (33.75)	934 (25.46)	
Unknown	1,466 (18.14)	893 (20.23)	573 (15.62)	
Marital Status, *n* (%)				0.879
Married	6,048 (74.82)	3,300 (74.75)	2,748 (74.92)	
Other	2,035 (25.18)	1,115 (25.25)	920 (25.08)	
Place of residence, *n* (%)				<0.001
Urban	2,287 (28.29)	1,642 (37.19)	645 (17.58)	
Rural	5,796 (71.71)	2,773 (62.81)	3,023 (82.42)	
Type of house structure, *n* (%)				<0.001
Reinforced concrete or masonry structure	6,904 (85.41)	4,061 (91.98)	2,843 (77.51)	
Others	1,179 (14.59)	354 (8.02)	825 (22.49)	
Income, *n* (%)				<0.001
<Mean	6,938 (85.83)	3,640 (82.45)	3,298 (89.91)	
≥Mean	1,085 (13.42)	741 (16.78)	344 (9.38)	
Unknown	60 (0.74)	34 (0.77)	26 (0.71)	
BMI, kg/m^2^, *n* (%)				<0.001
<24	3,680 (45.53)	1,891 (42.83)	1,789 (48.77)	
≥24	3,163 (39.13)	1,735 (39.30)	1,428 (38.93)	
Unknown	1,240 (15.34)	789 (17.87)	451 (12.30)	
Self-assessed health status, *n* (%)				<0.001
Very Good	465 (5.75)	271 (6.14)	194 (5.29)	
Good	520 (6.43)	311 (7.04)	209 (5.70)	
Fair	2,074 (25.66)	1,193 (27.02)	881 (24.02)	
Poor	823 (10.18)	395 (8.95)	428 (11.67)	
Very poor	213 (2.64)	80 (1.81)	133 (3.63)	
Unknown	3,988 (49.34)	2,165 (49.04)	1,823 (49.70)	
Night sleep duration, hours, *n* (%)				<0.001
7–9	3,451 (42.69)	1,866 (42.27)	1,585 (43.21)	
<7	4,175 (51.65)	2,336 (52.91)	1,839 (50.14)	
>9	371 (4.59)	180 (4.08)	191 (5.21)	
Unknown	86 (1.06)	33 (0.75)	53 (1.44)	
Smoking, *n* (%)				0.004
Never	4,526 (55.99)	2,540 (57.53)	1,986 (54.14)	
Former	1,131 (13.99)	613 (13.88)	518 (14.12)	
Now	2,426 (30.01)	1,262 (28.58)	1,164 (31.73)	
Drinking, *n* (%)				0.733
Never	594 (7.35)	320 (7.25)	274 (7.47)	
<1 time/month	694 (8.59)	370 (8.38)	324 (8.83)	
≥1 time/month	4,312 (53.35)	2,349 (53.20)	1,963 (53.52)	
Unknown	2,483 (30.72)	1,376 (31.17)	1,107 (30.18)	
Hypertension, *n* (%)				<0.001
No	3,814 (47.19)	2,068 (46.84)	1,746 (47.60)	
Yes	3,890 (48.13)	2,078 (47.07)	1,812 (49.40)	
Unknown	379 (4.69)	269 (6.09)	110 (3.00)	
Diabetes, *n* (%)				<0.001
No	6,240 (77.20)	3,297 (74.68)	2,943 (80.23)	
Yes	1,183 (14.64)	684 (15.49)	499 (13.60)	
Unknown	660 (8.17)	434 (9.83)	226 (6.16)	
Dyslipidemia, *n* (%)				<0.001
No	3,924 (48.55)	2,020 (45.75)	1,904 (51.91)	
Yes	3,474 (42.98)	1,945 (44.05)	1,529 (41.68)	
Unknown	685 (8.47)	450 (10.19)	235 (6.41)	
CVD, *n* (%)				<0.001
No	5,368 (66.41)	2,862 (64.82)	2,506 (68.32)	
Yes	780 (9.65)	365 (8.27)	415 (11.31)	
Unknown	1,935 (23.94)	1,188 (26.91)	747 (20.37)	
Chronic lung diseases, *n* (%)				<0.001
No	5,563 (68.82)	2,968 (67.23)	2,595 (70.75)	
Yes	573 (7.09)	256 (5.80)	317 (8.64)	
Unknown	1,947 (24.09)	1,191 (26.98)	756 (20.61)	
Arthritis or rheumatism, *n* (%)				<0.001
No	4,120 (50.97)	2,286 (51.78)	1,834 (50.00)	
Yes	2,022 (25.02)	939 (21.27)	1,083 (29.53)	
Unknown	1,941 (24.01)	1,190 (26.95)	751 (20.47)	
Cancer, *n* (%)				<0.001
No	6,074 (75.15)	3,187 (72.19)	2,887 (78.71)	
Yes	54 (0.67)	30 (0.68)	24 (0.65)	
Unknown	1,955 (24.19)	1,198 (27.13)	757 (20.64)	
Visual impairment (2015), *n* (%)				<0.001
No	5,889 (72.86)	3,323 (75.27)	2,566 (69.96)	
Yes	2,194 (27.14)	1,092 (24.73)	1,102 (30.04)	
Hearing impairment (2015), *n* (%)				0.267
No	7,689 (95.13)	4,211 (95.38)	3,478 (94.82)	
Yes	394 (4.87)	204 (4.62)	190 (5.18)	
Sensory disturbance (2015), *n* (%)				<0.001
Non-visual impairment and non-hearing impairment	5,495 (67.98)	3,119 (70.65)	2,376 (64.78)	
Visual impairment and non-hearing impairment	2,194 (27.14)	1,092 (24.73)	1,102 (30.04)	
Non-visual impairment and hearing impairment	394 (4.87)	204 (4.62)	190 (5.18)	
eGFR, mL/min/1.73m^2^, *n* (%)				<0.001
<60	215 (2.66)	138 (3.13)	77 (2.10)	
≥60	5,639 (69.76)	2,945 (66.70)	2,694 (73.45)	
Unknown	2,229 (27.58)	1,332 (30.17)	897 (24.45)	
Household fuels (2015 and 2018), *n* (%)				<0.001
Clean fuels and clean fuels	3,905 (48.31)	3,905 (88.45)	0 (0.00)	
Solid fuels and clean fuels	1,412 (17.47)	0 (0.00)	1,412 (38.50)	
Clean fuels and solid fuels	510 (6.31)	510 (11.55)	0 (0.00)	
Solid fuels and Solid fuels	2,256 (27.91)	0 (0.00)	2,256 (61.50)	
Hearing impairment (2018), *n* (%)				<0.001
No	7,197 (89.04)	3,996 (90.51)	3,201 (87.27)	
Yes	886 (10.96)	419 (9.49)	467 (12.73)	
Visual impairment (2018), *n* (%)				<0.001
No	5,722 (70.79)	3,224 (73.02)	2,498 (68.10)	
Yes	2,361 (29.21)	1,191 (26.98)	1,170 (31.90)	
DSI (2018), *n* (%)				<0.001
No	7,578 (93.75)	4,189 (94.88)	3,389 (92.39)	
Yes	505 (6.25)	226 (5.12)	279 (7.61)	

BMI, body mass index; CVD, cardiovascular disease; eGFR, estimated glomerular filtration rate; DSI, dual sensory impairment.

### Association between solid fuel use and DSI

The effect of household fuel use on DSI was presented in Table 2. In an analysis of baseline household fuel use, the use of solid fuels (OR = 1.53, 95%CI: 1.27–1.83) was linked to a higher risk of DSI compared to the use of clean fuels. After adjustment for age, gender, place of residence, and income, the use of solid fuels (OR = 1.23, 95%CI: 1.02–1.49) still increased the risk of DSI. After adjusting for all confounders (age, gender, place of residence, income, BMI, self-assessed health status, night sleep duration, diabetes, chronic lung diseases, and sensory disturbance), the use of solid fuels (*p* = 0.154) had no significant effect on DSI compared to the use of clean fuels.

**Table 2:** Association between household fuel use and the risk of DSI.

Variables	Unadjusted model		Model 1		Model 2	
	OR (95%CI)	*P*	OR (95%CI)	*P*	OR (95%CI)	*P*
Household fuels (2015)						
Clean fuels	Ref		Ref		Ref	
Solid fuels	1.53 (1.27–1.83)	<0.001	1.23 (1.02–1.49)	0.029	1.15 (0.95–1.40)	0.154
Household fuels (2015 and 2018)						
Clean fuels and clean fuels	Ref		Ref		Ref	
Solid fuels and clean fuels	1.24 (0.95–1.62)	0.115	1.06 (0.80–1.39)	0.693	0.99 (0.74–1.31)	0.925
Clean fuels and solid fuels	1.76 (1.24–2.50)	0.002	1.50 (1.05–2.15)	0.026	1.50 (1.04–2.16)	0.031
Solid fuels and Solid fuels	1.92 (1.56–2.37)	<0.001	1.48 (1.19–1.84)	<0.001	1.38 (1.10–1.73)	0.005

DSI, dual sensory impairment; OR, odds ratio; CI, confidence interval.

Unadjusted model was univariable logistic model.

Model 1 was multivariable logistic model adjusted for age, gender, place of residence, and income.

Model 2 was multivariable logistic model adjusted for age, gender, place of residence, income, BMI, self-assessed health status, night sleep duration, diabetes, chronic lung diseases, and sensory disturbance.

In an analysis of the impact of changes in household fuel use on DSI during the follow-up period, people in the clean fuels and solid fuels group (OR = 1.76, 95%CI: 1.24–2.50) and the solid fuels and solid fuels group (OR = 1.92, 95%CI: 1.56–2.37) had an increased risk of DSI compared to people in the clean fuels and clean fuels group, whereas there was no significant difference in the effect on DSI between people in the solid fuels and clean fuels group and the clean fuels and clean fuels group (*p* = 0.154). After adjustment for all confounders, people in the clean fuels and solid fuels group (OR = 1.50, 95%CI: 1.04–2.16) and the solid fuels and solid fuels group (OR = 1.38, 95%CI: 1.10–1.73) were still linked to higher risk of DSI, whereas no significant difference was observed in the effect on DSI between people in the solid fuels and clean fuels group and the clean fuels and clean fuels group (*p* = 0.99).

### Subgroup analyses on the association between solid fuel use and DSI

**Figure 2:**
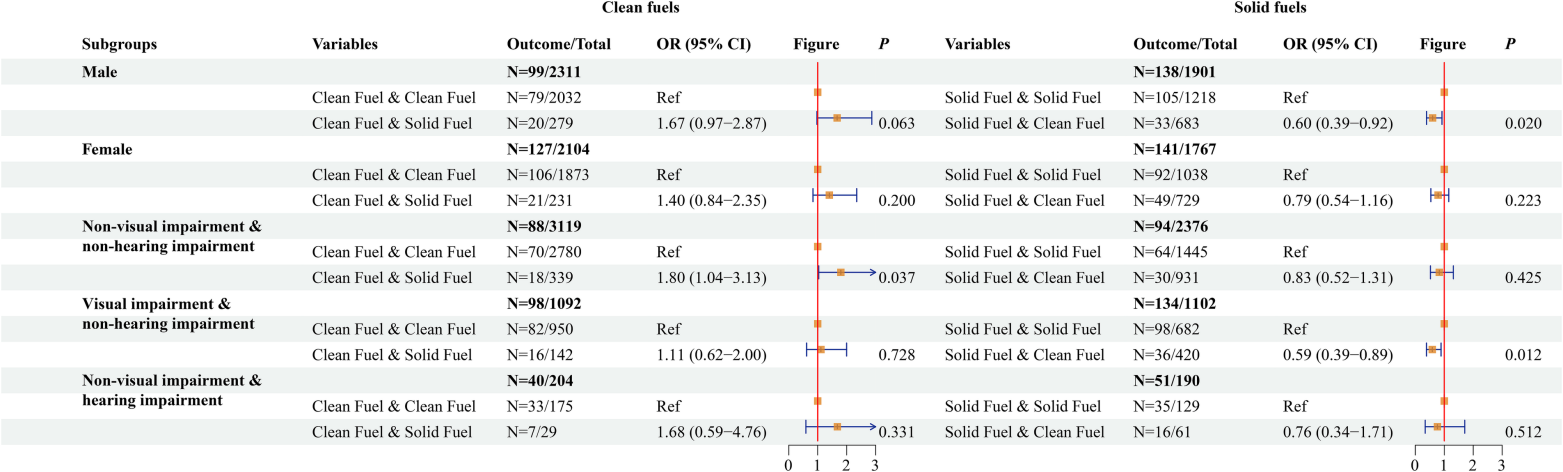
The impacts of changes in household fuel use on dual sensory impairment (DSI) during the follow-up period in different subgroups of the population.

The impacts of changes in household fuel use on DSI during the follow-up period in different subgroups of the population were shown in Figure 2. In males, switching to clean fuels during follow-up (OR = 0.60, 95%CI: 0.39–0.92) was associated with a lower risk of DSI among people who used solid fuels at baseline compared with those who remained on solid fuels use during follow-up, but no significant difference in the effect on DSI risk was found for switching to solid fuels during follow-up among participants who used clean fuels at baseline (*p* = 0.063). Among females, no effect of changes in clean or solid fuels use during follow-up on DSI risk was observed (*p* > 0.05). In people without hearing impairment and visual impairment at baseline, switching to solid fuels during follow-up (OR = 1.80, 95%CI: 1.04–3.13) increased the risk of DSI among people who used clean fuels at baseline compared with those who remained on clean fuels use during follow-up. Among people with visual impairment and non-hearing impairment at baseline, switching to clean fuels during follow-up (OR = 0.59, 95%CI: 0.39–0.89) decreased the risk of DSI among people who used solid fuels at baseline compared with those who remained on solid fuels use during follow-up.

## Discussion

This study assessed the effect of household fuel use on the risk of DSI in people 45 years and older. The use of solid fuels increased the risk of DSI compared to the use of clean fuels. Moreover, switching to solid fuels during follow-up (regardless of baseline solid or clean fuels) increased the risk of DSI compared with people who had been using clean fuels, whereas there was no significant difference in the effect on the risk of DSI between people who used solid fuels at baseline and clean fuels during follow-up and those who had been using clean fuels.

It has been reported that patients with DSI have an increased risk of cognitive impairment, dementia, and depression compared to patients with single visual impairment or hearing impairment (19, 20). In addition, patients with DSI are at higher risk for falls and unintentional injuries, and these may increase the risk of death (21, 22). Hearing impairments and visual impairments may accelerate cognitive decline, possibly due to the impact of sensory impairments on social isolation, depression, reduced physical and mental activity, and functional limitations (23). Our study explored the effect of household fuel use on the risk of DSI. The results showed that solid fuel use increased the risk of DSI compared to clean fuel use and that people who used solid fuels switched to clean fuel use during the study period reduced the risk of DSI. Several previous studies have also reported the effect of solid fuel use on sensory impairment (16, 17, 24). Cao et al. showed that household solid fuel use was associated with an increased risk of hearing damage (17). Cooking with solid fuels has been reported to increase the risk of distance visual impairment and near visual impairment (16). Household solid fuel use has been found to be a risk factor for the development of cognitive impairment (24). These studies all show the health hazards of household solid fuel use. The promotion of clean fuel use in households is important for health improvement.

The mechanism by which solid fuel use affects DSI is unclear. Some studies suggest that the effects of solid fuel use on visual impairment may be associated with household air pollution (HAP) (16, 24, 25). Environment PM from solid fuel use can cause oxidative stress and a decrease in endogenous antioxidants, which induces an inflammatory response and produces pro-inflammatory cytokines (26, 27). Fumes from solid fuels may also be deposited on the eyes, altering the chemical balance and immunity of the tear film, thereby increasing the risk of infection and directly damaging eye cells (14). Solid fuel use increases the risk of cataracts, which may be related to accelerated lens oxidation by free radicals in fumes (28). In addition, solid fuel use makes the eyes more susceptible to injury from sparks, wood chips, or sharp wood, which increases the risk of visual impairment due to eye trauma (16, 29). The effects of solid fuel use on hearing impairment may also be linked to HAP. Substances such as PM10, NO_2_, SO_2_, and CO produced by solid fuel use can promote oxidative stress and inflammation, which adversely affect cochlear endothelial cell function and microcirculation, and subsequently lead to hypoxia (30–33). Hypoxia damages cochlear cells and neurons in the inner ear and affects hearing function (33). Moreover, air pollution from the use of solid fuels may produce or act as reactive oxygen species, inducing mitochondrial damage and leading to inner ear hair cell death (34, 35).

This study analyzed the impact of household fuel use on DSI based on a large prospective population-based cohort. In addition, we considered the effect of changes in household fuel use types on DSI during the study period. Nevertheless, this study has several limitations. First, the diagnoses of hearing impairment and visual impairment were based on self-reported questionnaires. Since there are no records of relevant medical diagnoses in the CHARLS database, the use of self-reported diagnoses may be biased. The self-reporting data may not capture all relevant indoor air pollutants, which could affect the validity of our findings. Such as potential type of misclassification (probably non-differential) and direction of the bias (probably toward the null hypothesis), given the large sample size, this likelihood would be very low. Second, the level of exposure to household pollutants could not be accurately estimated for each participant due to the lack of relevant data. Third, the assessment of DSI at the end of follow-up and the limited time points for exposure changes may hinder our ability to definitively establish that exposure preceded outcome onset for all participants. This limitation could impact the interpretation of our results, particularly in terms of causality and the potential for reverse causation. Fourth, while the initial association was significant, the non-significant result after full adjustment indicates that other variables may indeed be influencing the relationship or over-adjustment. Although we have considered the effects of many confounders, there are still some potential confounders that may affect the results such as outdoor air quality, heat exposure, and noise.

## Conclusion

This study assessed the relationship between the type of household fuel use and the risk of DSI in a middle-aged and older adult Chinese population. Solid fuel use increased the risk of DSI, but subsequent switching to clean fuels by those using solid fuels reduced the risk of DSI. Promoting the use of clean fuels may be beneficial in reducing the burden of DSI.

## Data Availability

Publicly available datasets were analyzed in this study. This data can be found at: CHARLS database, http://charls.pku.edu.cn/.

## References

[1] Bourne RRA Flaxman SR Braithwaite T Cicinelli MV Das A Jonas JB. Magnitude, temporal trends, and projections of the global prevalence of blindness and distance and near vision impairment: a systematic review and meta-analysis. Lancet Glob Health. (2017) 5:e888–97. doi: 10.1016/s2214-109x(17)30293-0, PMID: 28779882

[2] Berk JB Stanton R Zechner J. Human capital, bankruptcy, and capital structure. J Finance. (2010) 65:891–926. doi: 10.1111/j.1540-6261.2010.01556.x

[3] Hsieh CT Klenow PJ. Misallocation and manufacturing Tfp in China and India. Q J Econ. (2009) 124:1403–48. doi: 10.1162/qjec.2009.124.4.1403

[4] Gopinath B Rochtchina E Wang JJ Schneider J Leeder SR Mitchell P. Prevalence of age-related hearing loss in older adults: Blue Mountains study. Arch Intern Med. (2009) 169:415–6. Epub 2009/02/25. doi: 10.1001/archinternmed.2008.597, PMID: 19237727

[5] Furtado JM. Vision loss in Australia by 2050. Clin Experiment Ophthalmol. (2020) 48:725–6. doi: 10.1111/ceo.13815, PMID: 32743863

[6] Chia EM Mitchell P Rochtchina E Foran S Golding M Wang JJ. Association between vision and hearing impairments and their combined effects on quality of life. Arch Ophthalmol. (2006) 124:1465–70. doi: 10.1001/archopht.124.10.1465, PMID: 17030715

[7] Wang Q Zhang S Wang Y Zhao D Zhou C. Dual sensory impairment as a predictor of loneliness and isolation in older adults: National Cohort Study. JMIR Public Health Surveill. (2022) 8:e39314. doi: 10.2196/39314, PMID: 36374533 PMC9706378

[8] Deardorff WJ Liu PL Sloane R Van Houtven C Pieper CF Hastings SN. Association of Sensory and Cognitive Impairment with healthcare utilization and cost in older adults. J Am Geriatr Soc. (2019) 67:1617–24. doi: 10.1111/jgs.15891, PMID: 30924932 PMC6684393

[9] Bruce N Perez-Padilla R Albalak R. Indoor air pollution in developing countries: a major environmental and public health challenge. Bull World Health Organ. (2000) 78:1078–92. 11019457 PMC2560841

[10] Gall ET Carter EM Earnest CM Stephens B. Indoor air pollution in developing countries: research and implementation needs for improvements in global public health. Am J Public Health. (2013) 103:e67. doi: 10.2105/ajph.2012.300955, PMID: 23409891 PMC3673244

[11] Bonjour S Adair-Rohani H Wolf J Bruce NG Mehta S Prüss-Ustün A. Solid fuel use for household cooking: country and regional estimates for 1980-2010. Environ Health Perspect. (2013) 121:784–90. doi: 10.1289/ehp.1205987, PMID: 23674502 PMC3701999

[12] Chang KH Tsai SC Lee CY Chou RH Fan HC Lin FC. Increased risk of sensorineural hearing loss as a result of exposure to air pollution. Int J Environ Res Public Health. (2020) 17:1969. doi: 10.3390/ijerph17061969, PMID: 32192124 PMC7143358

[13] Choi HG Min C Kim SY. Air pollution increases the risk of Ssnhl: a nested case-control study using meteorological data and National Sample Cohort Data. Sci Rep. (2019) 9:8270. doi: 10.1038/s41598-019-44618-0, PMID: 31164673 PMC6547844

[14] Karakoçak BB Patel S Ravi N Biswas P. Investigating the effects of stove emissions on ocular and Cancer cells. Sci Rep. (2019) 9:1870. doi: 10.1038/s41598-019-38803-4, PMID: 30755694 PMC6372759

[15] Zhao Y Hu Y Smith JP Strauss J Yang G. Cohort profile: the China health and retirement longitudinal study (Charls). Int J Epidemiol. (2014) 43:61–8. doi: 10.1093/ije/dys203, PMID: 23243115 PMC3937970

[16] Jiang Q Wang S Zhang H Guo Y Lou Y Huang S. The association between solid fuel use and visual impairment among middle-aged and older Chinese adults: Nationwide population-based cohort study. JMIR Public Health Surveill. (2023) 9:e43914. doi: 10.2196/43914, PMID: 37494091 PMC10413239

[17] Liu T Cao L Lv P Bai S. Associations between household solid fuel use and hearing loss in a Chinese population: a population-based prospective cohort study. Ecotoxicol Environ Saf. (2022) 236:113506. doi: 10.1016/j.ecoenv.2022.113506, PMID: 35421824

[18] Zhu J Wen C Zhu J Zhang H Wang X. A polynomial algorithm for best-subset selection problem. Proc Natl Acad Sci USA. (2020) 117:33117–23. doi: 10.1073/pnas.2014241117, PMID: 33328272 PMC7777147

[19] Kuo PL Huang AR Ehrlich JR Kasper J Lin FR McKee MM. Prevalence of concurrent functional vision and hearing impairment and association with dementia in community-dwelling Medicare beneficiaries. JAMA Netw Open. (2021) 4:e211558. doi: 10.1001/jamanetworkopen.2021.1558, PMID: 33739429 PMC8601132

[20] Rong H Lai X Jing R Wang X Fang H Mahmoudi E. Association of Sensory Impairments with cognitive decline and depression among older adults in China. JAMA Netw Open. (2020) 3:e2014186. doi: 10.1001/jamanetworkopen.2020.14186, PMID: 32990739 PMC7525357

[21] Gopinath B McMahon CM Burlutsky G Mitchell P. Hearing and vision impairment and the 5-year incidence of falls in older adults. Age Ageing. (2016) 45:409–14. doi: 10.1093/ageing/afw022, PMID: 26946051

[22] Assi L Shakarchi AF Sheehan OC Deal JA Swenor BK Reed NS. Assessment of sensory impairment and health care satisfaction among Medicare beneficiaries. JAMA Netw Open. (2020) 3:e2025522. doi: 10.1001/jamanetworkopen.2020.25522, PMID: 33185678 PMC7666423

[23] Fischer ME Cruickshanks KJ Klein BE Klein R Schubert CR Wiley TL. Multiple sensory impairment and quality of life. Ophthalmic Epidemiol. (2009) 16:346–53. doi: 10.3109/09286580903312236, PMID: 19995199 PMC2805084

[24] Li S Cui G Hu M Hu Y Ren L Jiang Y. Associations between cooking fuel use, its transitions, and worsening sensory impairments among Chinese middle-aged and older adults: a cohort study. BMC Geriatr. (2024) 24:288. doi: 10.1186/s12877-024-04746-3, PMID: 38539094 PMC10976684

[25] Chan KH Yan M Bennett DA Guo Y Chen Y Yang L. Long-term solid fuel use and risks of major eye diseases in China: a population-based cohort study of 486,532 adults. PLoS Med. (2021) 18:e1003716. doi: 10.1371/journal.pmed.1003716, PMID: 34324491 PMC8321372

[26] Ghio AJ Carraway MS Madden MC. Composition of air pollution particles and oxidative stress in cells, tissues, and living systems. J Toxicol Environ Health B Crit Rev. (2012) 15:1–21. doi: 10.1080/10937404.2012.632359, PMID: 22202227

[27] Hirota JA Hirota SA Warner SM Stefanowicz D Shaheen F Beck PL. The airway epithelium nucleotide-binding domain and leucine-rich repeat protein 3 Inflammasome is activated by urban particulate matter. J Allergy Clin Immunol. (2012) 129:1116–25.e6. doi: 10.1016/j.jaci.2011.11.033, PMID: 22227418

[28] Vashist P Tandon R Murthy GVS Barua CK Deka D Singh S. Association of cataract and sun exposure in geographically diverse populations of India: the case study. First report of the Icmr-eye see study group. PLoS One. (2020) 15:e0227868. doi: 10.1371/journal.pone.0227868, PMID: 31971985 PMC6977762

[29] Xiang P Liu RY Sun HJ Han YH He RW Cui XY. Molecular mechanisms of dust-induced toxicity in human corneal epithelial cells: water and organic extract of office and house dust. Environ Int. (2016) 92-93:348–56. Epub 2016/05/01. doi: 10.1016/j.envint.2016.04.013, PMID: 27131017

[30] Li W Wilker EH Dorans KS Rice MB Schwartz J Coull BA. Short-term exposure to air pollution and biomarkers of oxidative stress: the Framingham heart study. J Am Heart Assoc. (2016) 5:e002742. doi: 10.1161/jaha.115.002742, PMID: 27126478 PMC4889166

[31] Pope CA 3rd Bhatnagar A McCracken JP Abplanalp W Conklin DJ O'Toole T. Exposure to fine particulate air pollution is associated with endothelial injury and systemic inflammation. Circ Res. (2016) 119:1204–14. doi: 10.1161/circresaha.116.309279, PMID: 27780829 PMC5215745

[32] Migliore L Coppedè F. Environmental-induced oxidative stress in neurodegenerative disorders and aging. Mutat Res. (2009) 674:73–84. Epub 2008/10/28. doi: 10.1016/j.mrgentox.2008.09.013, PMID: 18952194

[33] Olivetto E Simoni E Guaran V Astolfi L Martini A. Sensorineural hearing loss and ischemic injury: development of animal models to assess vascular and oxidative effects. Hear Res. (2015) 327:58–68. doi: 10.1016/j.heares.2015.05.004, PMID: 25987500

[34] Kelly FJ. Oxidative stress: its role in air pollution and adverse health effects. Occup Environ Med. (2003) 60:612–6. doi: 10.1136/oem.60.8.612, PMID: 12883027 PMC1740593

[35] Lyu AR Kim TH Park SJ Shin SA Jeong SH Yu Y. Mitochondrial damage and necroptosis in aging cochlea. Int J Mol Sci. (2020) 21:2505. doi: 10.3390/ijms21072505, PMID: 32260310 PMC7177801

