# Supplementary material for: Association between household solid fuel use and dual sensory impairment in a Chinese population: a retrospective cohort study
Source: Front Public Health. 2025 Jun 10;13:1439673. doi: 10.3389/fpubh.2025.1439673 (PMC12185414; doi:10.3389/fpubh.2025.1439673)
Supplement: Supplementary file 1 [file Table_1.docx]

**Supplement Tabel 1**. The optimum subset of covariates and their coefficients screened by the Adaptive Best-Subset Selection (ABESS) method.

| **Variables** | ***β*** |
| --- | --- |
| Age | 0.03532487 |
| Gender |  |
| Male | Ref |
| Female | 0.25217473 |
| Place of residence |  |
| Urban | Ref |
| Rural | 0.60757762 |
| Income |  |
| <Mean | Ref |
| ≥Mean | -0.50689821 |
| Unknown | - |
| Body mass index (BMI) |  |
| <24kg/m^2^ | Ref |
| ≥24kg/m^2^ | -0.30748769 |
| Unknown | - |
| Self-assessed health status |  |
| Very Good | Ref |
| Good | - |
| Fair | - |
| Poor | 0.39496558 |
| Very poor | 0.83824241 |
| Unknown | - |
| Night sleep duration |  |
| 7-9 | Ref |
| <7 | 0.23107096 |
| >9 | 0.57224909 |
| Unknown | - |
| Diabetes |  |
| No | Ref |
| Yes | 0.28581471 |
| Unknown | - |
| Chronic lung diseases |  |
| No | Ref |
| Yes | 0.34770100 |
| Unknown | - |
| Sensory disturbance (2015) |  |
| Non-visual impairment & non-hearing impairment | Ref |
| Visual impairment & non-hearing impairment | 1.07543852 |
| Non-visual impairment & hearing impairment | 1.85092351 |

**Supplement Tabel 2**. Multicollinearity test between variables using variance inflation factor (VIF).

| **Variables** | **Household fuels (2015) model** | | | **Household fuels (2015 & 2018) model** | | |
| --- | --- | --- | --- | --- | --- | --- |
|  | **VIF** | **Df** | **VIF^[1/(2*Df)]** | **VIF** | **Df** | **VIF^[1/(2*Df)]** |
| Household fuels/household fuels (2015 & 2018) | 1.067548 | 1 | 1.033222 | 1.097607 | 3 | 1.015643 |
| Age | 1.146811 | 1 | 1.070892 | 1.143642 | 1 | 1.069412 |
| Gender | 1.065315 | 1 | 1.032141 | 1.069657 | 1 | 1.034242 |
| Place of residence | 1.098318 | 1 | 1.048007 | 1.114768 | 1 | 1.055826 |
| Income | 1.063126 | 2 | 1.015421 | 1.064266 | 2 | 1.015693 |
| BMI | 1.207494 | 2 | 1.048265 | 1.208257 | 2 | 1.048431 |
| Self-assessed health status | 1.065503 | 5 | 1.006365 | 1.066535 | 5 | 1.006462 |
| Night sleep duration | 1.063274 | 3 | 1.010278 | 1.063663 | 3 | 1.010339 |
| Diabetes | 1.466641 | 2 | 1.100477 | 1.469123 | 2 | 1.100942 |
| Chronic lung diseases | 1.375036 | 2 | 1.082875 | 1.377640 | 2 | 1.083388 |
| Sensory disturbance (2015) | 1.061010 | 2 | 1.014915 | 1.059624 | 2 | 1.014584 |

Note: BMI, body mass index.

**Supplement Tabel 3**. The characteristics of participants based on household fuel use at baseline and during follow-up.

| **Variables** | **Household fuels (baseline & follow-up)** | | | | | | | |
| --- | --- | --- | --- | --- | --- | --- | --- | --- |
|  | **Total (N=4415)** | **Clean fuels & clean fuels (N=3905)** | **Clean fuels & solid fuels (N=510)** | ***P*** | **Total (N=3668)** | **Solid fuels & clean fuels (N=1412)** | **Solid fuels & solid fuels (N=2256)** | ***P*** |
| Age, years, Mean (±SD) | 59.67 (±9.78) | 59.49 (±9.76) | 61.06 (±9.83) | 0.001 | 61.27 (±9.73) | 60.66 (±10.00) | 61.64 (±9.55) | 0.003 |
| Gender, n (%) |  |  |  | 0.276 |  |  |  | 0.001 |
| Male | 2311 (52.34) | 2032 (52.04) | 279 (54.71) |  | 1901 (51.83) | 683 (48.37) | 1218 (53.99) |  |
| Female | 2104 (47.66) | 1873 (47.96) | 231 (45.29) |  | 1767 (48.17) | 729 (51.63) | 1038 (46.01) |  |
| Education, n (%) |  |  |  | 0.001 |  |  |  | <0.001 |
| Illiterate | 590 (13.36) | 504 (12.91) | 86 (16.86) |  | 780 (21.26) | 261 (18.48) | 519 (23.01) |  |
| Primary school or below | 1442 (32.66) | 1253 (32.09) | 189 (37.06) |  | 1381 (37.65) | 475 (33.64) | 906 (40.16) |  |
| Middle school or above | 1490 (33.75) | 1339 (34.29) | 151 (29.61) |  | 934 (25.46) | 423 (29.96) | 511 (22.65) |  |
| Unknown | 893 (20.23) | 809 (20.72) | 84 (16.47) |  | 573 (15.62) | 253 (17.92) | 320 (14.18) |  |
| Marital status, n (%) |  |  |  | 0.043 |  |  |  | 0.003 |
| Married | 3300 (74.75) | 2938 (75.24) | 362 (70.98) |  | 2748 (74.92) | 1019 (72.17) | 1729 (76.64) |  |
| Other | 1115 (25.25) | 967 (24.76) | 148 (29.02) |  | 920 (25.08) | 393 (27.83) | 527 (23.36) |  |
| Place of residence, n (%) |  |  |  | <0.001 |  |  |  | <0.001 |
| Urban | 1642 (37.19) | 1514 (38.77) | 128 (25.10) |  | 645 (17.58) | 391 (27.69) | 254 (11.26) |  |
| Rural | 2773 (62.81) | 2391 (61.23) | 382 (74.90) |  | 3023 (82.42) | 1021 (72.31) | 2002 (88.74) |  |
| Type of house structure, n (%) |  |  |  | <0.001 |  |  |  | <0.001 |
| Reinforced concrete or masonry structure | 4061 (91.98) | 3643 (93.29) | 418 (81.96) |  | 2843 (77.51) | 1225 (86.76) | 1618 (71.72) |  |
| Others | 354 (8.02) | 262 (6.71) | 92 (18.04) |  | 825 (22.49) | 187 (13.24) | 638 (28.28) |  |
| Income, n (%) |  |  |  | 0.104 |  |  |  | 0.011 |
| <Mean | 3640 (82.45) | 3203 (82.02) | 437 (85.69) |  | 3298 (89.91) | 1243 (88.03) | 2055 (91.09) |  |
| ≥Mean | 741 (16.78) | 672 (17.21) | 69 (13.53) |  | 344 (9.38) | 158 (11.19) | 186 (8.24) |  |
| Unknown | 34 (0.77) | 30 (0.77) | 4 (0.78) |  | 26 (0.71) | 11 (0.78) | 15 (0.66) |  |
| BMI, kg/m^2^, n (%) |  |  |  | 0.018 |  |  |  | <0.001 |
| <24 | 1891 (42.83) | 1644 (42.10) | 247 (48.43) |  | 1789 (48.77) | 611 (43.27) | 1178 (52.22) |  |
| ≥24 | 1735 (39.30) | 1548 (39.64) | 187 (36.67) |  | 1428 (38.93) | 599 (42.42) | 829 (36.75) |  |
| Unknown | 789 (17.87) | 713 (18.26) | 76 (14.90) |  | 451 (12.30) | 202 (14.31) | 249 (11.04) |  |
| Self-assessed health status, n (%) |  |  |  | 0.243 |  |  |  | 0.305 |
| Very Good | 271 (6.14) | 246 (6.30) | 25 (4.90) |  | 194 (5.29) | 87 (6.16) | 107 (4.74) |  |
| Good | 311 (7.04) | 282 (7.22) | 29 (5.69) |  | 209 (5.70) | 86 (6.09) | 123 (5.45) |  |
| Fair | 1193 (27.02) | 1061 (27.17) | 132 (25.88) |  | 881 (24.02) | 348 (24.65) | 533 (23.63) |  |
| Poor | 395 (8.95) | 338 (8.66) | 57 (11.18) |  | 428 (11.67) | 166 (11.76) | 262 (11.61) |  |
| Very poor | 80 (1.81) | 71 (1.82) | 9 (1.76) |  | 133 (3.63) | 49 (3.47) | 84 (3.72) |  |
| Unknown | 2165 (49.04) | 1907 (48.83) | 258 (50.59) |  | 1823 (49.70) | 676 (47.88) | 1147 (50.84) |  |
| Night sleep duration, hours, n (%) |  |  |  | 0.913 |  |  |  | 0.969 |
| 7-9 | 1866 (42.27) | 1644 (42.10) | 222 (43.53) |  | 1585 (43.21) | 614 (43.48) | 971 (43.04) |  |
| <7 | 2336 (52.91) | 2073 (53.09) | 263 (51.57) |  | 1839 (50.14) | 707 (50.07) | 1132 (50.18) |  |
| >9 | 180 (4.08) | 159 (4.07) | 21 (4.12) |  | 191 (5.21) | 72 (5.10) | 119 (5.27) |  |
| Unknown | 33 (0.75) | 29 (0.74) | 4 (0.78) |  | 53 (1.44) | 19 (1.35) | 34 (1.51) |  |
| Smoking, n (%) |  |  |  | 0.078 |  |  |  | 0.027 |
| Never | 2540 (57.53) | 2267 (58.05) | 273 (53.53) |  | 1986 (54.14) | 804 (56.94) | 1182 (52.39) |  |
| Former | 613 (13.88) | 543 (13.91) | 70 (13.73) |  | 518 (14.12) | 188 (13.31) | 330 (14.63) |  |
| Now | 1262 (28.58) | 1095 (28.04) | 167 (32.75) |  | 1164 (31.73) | 420 (29.75) | 744 (32.98) |  |
| Drinking, n (%) |  |  |  | 0.146 |  |  |  | 0.064 |
| Never | 320 (7.25) | 280 (7.17) | 40 (7.84) |  | 274 (7.47) | 89 (6.30) | 185 (8.20) |  |
| <1 time/month | 370 (8.38) | 316 (8.09) | 54 (10.59) |  | 324 (8.83) | 134 (9.49) | 190 (8.42) |  |
| ≥1 time/month | 2349 (53.20) | 2097 (53.70) | 252 (49.41) |  | 1963 (53.52) | 778 (55.10) | 1185 (52.53) |  |
| Unknown | 1376 (31.17) | 1212 (31.04) | 164 (32.16) |  | 1107 (30.18) | 411 (29.11) | 696 (30.85) |  |
| Hypertension, n (%) |  |  |  | 0.036 |  |  |  | 0.522 |
| No | 2068 (46.84) | 1851 (47.40) | 217 (42.55) |  | 1746 (47.60) | 672 (47.59) | 1074 (47.61) |  |
| Yes | 2078 (47.07) | 1811 (46.38) | 267 (52.35) |  | 1812 (49.40) | 692 (49.01) | 1120 (49.65) |  |
| Unknown | 269 (6.09) | 243 (6.22) | 26 (5.10) |  | 110 (3.00) | 48 (3.40) | 62 (2.75) |  |
| Diabetes, n (%) |  |  |  | 0.716 |  |  |  | 0.138 |
| No | 3297 (74.68) | 2909 (74.49) | 388 (76.08) |  | 2943 (80.23) | 1111 (78.68) | 1832 (81.21) |  |
| Yes | 684 (15.49) | 608 (15.57) | 76 (14.90) |  | 499 (13.60) | 203 (14.38) | 296 (13.12) |  |
| Unknown | 434 (9.83) | 388 (9.94) | 46 (9.02) |  | 226 (6.16) | 98 (6.94) | 128 (5.67) |  |
| Dyslipidemia, n (%) |  |  |  | 0.476 |  |  |  | 0.102 |
| No | 2020 (45.75) | 1774 (45.43) | 246 (48.24) |  | 1904 (51.91) | 703 (49.79) | 1201 (53.24) |  |
| Yes | 1945 (44.05) | 1732 (44.35) | 213 (41.76) |  | 1529 (41.68) | 610 (43.20) | 919 (40.74) |  |
| Unknown | 450 (10.19) | 399 (10.22) | 51 (10.00) |  | 235 (6.41) | 99 (7.01) | 136 (6.03) |  |
| CVD, n (%) |  |  |  | 0.007 |  |  |  | 0.027 |
| No | 2862 (64.82) | 2528 (64.74) | 334 (65.49) |  | 2506 (68.32) | 930 (65.86) | 1576 (69.86) |  |
| Yes | 365 (8.27) | 307 (7.86) | 58 (11.37) |  | 415 (11.31) | 165 (11.69) | 250 (11.08) |  |
| Unknown | 1188 (26.91) | 1070 (27.40) | 118 (23.14) |  | 747 (20.37) | 317 (22.45) | 430 (19.06) |  |
| Chronic lung diseases, n (%) |  |  |  | 0.023 |  |  |  | 0.042 |
| No | 2968 (67.23) | 2616 (66.99) | 352 (69.02) |  | 2595 (70.75) | 973 (68.91) | 1622 (71.90) |  |
| Yes | 256 (5.80) | 216 (5.53) | 40 (7.84) |  | 317 (8.64) | 118 (8.36) | 199 (8.82) |  |
| Unknown | 1191 (26.98) | 1073 (27.48) | 118 (23.14) |  | 756 (20.61) | 321 (22.73) | 435 (19.28) |  |
| Cancer, n (%) |  |  |  | 0.134 |  |  |  | 0.022 |
| No | 3187 (72.19) | 2801 (71.73) | 386 (75.69) |  | 2887 (78.71) | 1078 (76.35) | 1809 (80.19) |  |
| Yes | 30 (0.68) | 26 (0.67) | 4 (0.78) |  | 24 (0.65) | 10 (0.71) | 14 (0.62) |  |
| Unknown | 1198 (27.13) | 1078 (27.61) | 120 (23.53) |  | 757 (20.64) | 324 (22.95) | 433 (19.19) |  |
| Sensory disturbance, n (%) |  |  |  | 0.078 |  |  |  | 0.148 |
| Non-visual impairment & non-hearing impairment | 3119 (70.65) | 2780 (71.19) | 339 (66.47) |  | 2376 (64.78) | 931 (65.93) | 1445 (64.05) |  |
| Visual impairment & non-hearing impairment | 1092 (24.73) | 950 (24.33) | 142 (27.84) |  | 1102 (30.04) | 420 (29.75) | 682 (30.23) |  |
| Non-visual impairment & hearing impairment | 204 (4.62) | 175 (4.48) | 29 (5.69) |  | 190 (5.18) | 61 (4.32) | 129 (5.72) |  |
| eGFR, mL/min/1.73m², n (%) |  |  |  | 0.347 |  |  |  | 0.218 |
| <60 | 138 (3.13) | 125 (3.20) | 13 (2.55) |  | 77 (2.10) | 34 (2.41) | 43 (1.91) |  |
| ≥60 | 2945 (66.70) | 2591 (66.35) | 354 (69.41) |  | 2694 (73.45) | 1016 (71.95) | 1678 (74.38) |  |
| Unknown | 1332 (30.17) | 1189 (30.45) | 143 (28.04) |  | 897 (24.45) | 362 (25.64) | 535 (23.71) |  |
| DSI (2018), n (%) |  |  |  | 0.002 |  |  |  | 0.001 |
| No | 4189 (94.88) | 3720 (95.26) | 469 (91.96) |  | 3389 (92.39) | 1330 (94.19) | 2059 (91.27) |  |
| Yes | 226 (5.12) | 185 (4.74) | 41 (8.04) |  | 279 (7.61) | 82 (5.81) | 197 (8.73) |  |

Note: BMI, body mass index; CVD, cardiovascular disease; eGFR, estimated glomerular filtration rate; DSI, dual sensory impairment.
